# Milk consumption and childhood anthropometric failure in India: Analysis of a national survey

**DOI:** 10.1111/mcn.13090

**Published:** 2020-09-30

**Authors:** Shelley M. Vanderhout, Daniel J. Corsi

**Affiliations:** ^1^ Department of Nutritional Sciences University of Toronto Toronto Ontario Canada; ^2^ School of Epidemiology and Public Health University of Ottawa Ottawa Ontario Canada

**Keywords:** anthropometric failure, children, dairy, India, milk, stunting, underweight

## Abstract

Dairy milk has been shown to contribute to child growth in many countries, but the relationship between milk intake and anthropometric outcomes among Indian children has not been studied. The objectives were to describe children aged 6–59 months who consume dairy milk in India and determine if dairy milk consumption was associated with lower odds of stunting, underweight and anthropometric failure among Indian children. This was a cross‐sectional study based on the fourth Indian National Family Health Survey (NFHS‐4), which was a national survey conducted between 2015 and 2016 by the Ministry of Health and Family Welfare. The primary exposure was the consumption of dairy milk within the past day or night. The primary outcomes were stunting (height‐for‐age *z* score < −2), underweight (weight‐for‐age *z* score < −2) and the composite index of anthropometric failure (CIAF), which is a combination of weight‐for‐age, weight‐for‐height and height‐for‐age. Multivariable logistic regression models and coarsened exact matching (CEM) were used to determine the relationship between dairy milk and odds ratios of each outcome. Setting was in India. Participants were children (*N* = 107,639) aged 6–59 months. Children who consumed dairy milk in the past day or night had an odds ratio of 0.95 for underweight (95% CI 0.92–0.98, *P* = .0005), 0.93 for stunting (95% CI 0.90–0.96, *P* < .0001) and 0.96 for CIAF (95% CI 0.93–0.99, *P* = .004), compared with children who did not consume dairy milk after adjusting for relevant covariates. When CEM was used among a subset (*n* = 28,207), evidence for relationships between dairy milk and anthropometric outcomes was consistent but slightly weaker. Widespread, equitable access to dairy milk among childhood may be part of an effort to lower the risk of anthropometric failure among children in India.

**Abbreviations**
BMI: body mass index
CEM: coarsened exact matching
CIAF: composite index of anthropometric failure
NFHS: National Family Health Survey
WHO: World Health Organisation

**Key messages**

- Dairy milk is known to provide essential nutrients for growth during childhood, can be a vehicle for micronutrient supplementation, and is becoming more widely available in India.
- In this study, we identified that children aged 6–59 months in India who consumed milk had lower odds of stunting, underweight and anthropometric failure after adjustment for relevant covariates.
- Efforts to improve children's nutritional status in India may include better access to safe, sterile dairy milk.

## 1 BACKGROUND

Children in India under 5 years old represent 31% of children with stunting and 24% of undernourished children worldwide. In 2017, 68% of deaths among Indian children under age 5 years were attributable to malnutrition (India State‐Level Disease Burden Initiative Malnutrition C, 2019). Undernutrition leading to stunting and underweight in childhood has been linked to irreversible, lasting effects(Caulfield, Richard, & Black, 2004) such as lower cognitive and motor performance (Grantham‐McGregor et al., 2007) and higher mortality (Pelletier & Frongillo, 2003). A variety of factors including socio‐economic status (Agrawal et al., 2019), household food insecurity (Chandrasekhar, Aguayo, Krishna, & Nair, 2017), parental education, dietary intake and anthropometrics (Aguayo, Nair, Badgaiyan, & Krishna, 2016; Corsi, Mejia‐Guevara, & Subramanian, 2016) and dietary diversity (Chandrasekhar et al., 2017; Corsi et al., 2016) are known to influence a child's risk of anthropometric failure in India. Undernutrition leading to poor growth is not just a childhood problem; it extends into adulthood and to future generations such that parents who are stunted are more likely to have children with stunting (Corsi et al., 2016). On the other hand, effective nutritional interventions to reduce child stunting can also benefit subsequent offspring growth (Martorell & Zongrone, 2012).

India has a history of an unestablished dairy industry and lack of access to safe, inexpensive milk (Ohlan, 2016). However, 30–50% of children in India consume dairy milk (Agrawal et al., 2019), which has been associated with taller height (de Beer, 2012) and lower risk of undernutrition (Basit, Nair, Chakraborthy, Darshan, & Kamath, 2012; Dror & Allen, 2011). The Infant and Young Child Feeding Practices (Ministry of Women and Child Development (Food and Nutrition Board), 2006) for India and Indian Dietary Guidelines (National Institute of Nutrition, 2011) suggest exclusive breastfeeding during the first 6 months of life and introduction of complementary foods including dairy thereafter. Dairy milk is a nutrient‐rich food well accepted by children, providing energy, protein, fat, vitamin B_12_, calcium, and can be fortified with vitamins A and D and other micronutrients vital to child growth and development (Dror & Allen, 2011). Though dairy milk consumption and child growth have been studied in developed countries (de Beer, 2012), the specific relationship between dairy milk and child stunting and underweight within the context of Indian dietary intakes is not well described (Shivakumar et al., 2019). As dairy milk has become more widely available in India in recent years (Gupta, 2015; National Institute of Nutrition, 2011), it may have unrealized potential to address undernutrition among Indian children.

In this research, we address the need to investigate the relationship between dairy milk consumption and child stunting and underweight in India using a large‐scale nationally representative survey. The objectives of this study were to describe children aged 6–59 months who consume dairy milk in India and determine if dairy milk consumption was associated with lower odds of stunting, underweight and anthropometric failure among Indian children. It was hypothesised that higher dairy milk consumption during childhood in India was associated with lower odds of stunting, underweight or anthropometric failure.

## 2 METHODS

We conducted a cross‐sectional study using the fourth Indian National Family Health Survey (NFHS‐4), a national survey conducted in India between 2015 and 2016 by the Ministry of Health and Family Welfare (International Institute for Population Sciences (IIPS) and ICF, 2017). NFHS‐4 wa intended to represent the population of reproductive‐aged women (15–49 years) and collected information on socio‐demographic characteristics, household water and sanitation, child health, women and men's health and other related variables. The NFHS‐4 was a two‐stage stratified survey designed to be nationally representative of the household population of women aged 15–49 years and covered all states and union territories in India (IIPS and ICF, 2017) and used the 2011 Census of India as the sampling frame. Urban and rural areas were sampled separately with additional stratification in rural areas based on the proportion of Scheduled Castes and Scheduled Tribes. Primary sampling units (PSUs) were defined as census enumeration blocks in urban areas and villages in rural areas, which typically contain 100–150 households each, and selected with a probability proportional to size within each stratum. Selected PSUs were visited by field teams who compiled lists of all residential households to serve as the sampling frame for the second survey stage. A fixed number of 22 households were then randomly selected within PSU to be visited by survey teams (IIPS and ICF, 2017).

Survey respondents provided informed oral consent prior to each interview. Questionnaires were administered orally by interviewers and responses recorded using electronic data capture and CAPI software to provide feedback and ensure the robustness of data quality. Fieldwork was completed between January 20, 2015 and December 4, 2016. The survey response rate was nearly 98% at the household level and was 97% among eligible women.

### 2.1 Eligibility criteria

The total study sample was composed of singleton children aged 6–59 months at the time of the NFHS‐4 survey (*N* = 107,639). Cases with missing outcome or exposure data were excluded.

### 2.2 Exposures

The primary exposure was child consumption of tinned, powdered or fresh milk in the day or night preceding the interview, measured as a dichotomous variable (yes/no). When powdered and tinned milk is prepared according to directions, the nutritional content is analogous to fresh milk for macronutrients and most micronutrients (Dietitians of Canada, 2020a; Dietitians of Canada, 2020b). Factors hypothesised to have a relationship with both the exposure and outcome included child age, household wealth (measured in quintiles; Rutstein & Johnson, 2004), maternal education (measured as none, primary, secondary or higher), maternal body mass index (BMI, measured as weight in kilograms divided by height in m^2^), birth weight in kilogrammes, birth size (used as a proxy when birth weight is not possible to measure) (Dharmalingam, Navaneetham, & Krishnakumar, 2010), time of breastfeeding initiation after birth (measured in hours), current breastfeeding (yes or no), fever or cough in past 2 weeks (yes or no), home air quality related to cooking fuels used (clean or solid), access to an improved sanitary facility and drinking water source (yes or no), safe disposal of stools (yes or no), child vaccination status (complete or incomplete), vitamin A supplementation in the past 6 months (yes or no), dietary diversity score and state of residence. Many of these factors describe living environments patterned by socio‐economic status; they are related to adequate and safe food, food handling, storage and preparation procedures in the home, which can influence child growth and anthropometry.

Dietary diversity was calculated as a score from 0 to 7 points (World Health Organization, 2010). If a child consumed the following foods during the preceding day or night to the interview, 1 point was given for at least one consumption of dairy other than milk including yogurt and cheese; chicken, duck, other birds *or* liver, heart or organ meat *or* fish or shellfish *or* other meat; eggs; peas, beans, lentils or nuts; breads, noodles or grains *or* potatoes, cassava or tubers; pumpkin, carrot or squash *or* mango, papaya or vitamin A‐containing fruit; and dark green leafy vegetables *or* other fruit. Dietary diversity scores were classified into quintiles for the child's age group (<12; 12–24; 24–36; 36–48; and >48 months), as diet varies during different stages of early childhood and to capture the high proportion of children with low dietary diversity in the sample. Quintiles were made within each age group and then summed and reported among the entire sample.

### 2.3 Outcomes

The primary outcomes were child stunting, underweight and composite index of anthropometric failure, measured by height‐for‐age (HAZ), weight‐for‐age (WAZ) and weight‐for‐height *z* scores, which were standardised according to the World Health Organisation (WHO) Growth Standards (World Health Organization, 1995). Stunting was defined as HAZ less than 2 standard deviations (*SD*) below the WHO Growth Standards median and underweight as WAZ less than 2 *SD* below the median (World Health Organization, 2010). The composite index for anthropometric failure (CIAF) combines WAZ, weight‐for‐height and HAZ to create a single measure of child anthropometry related to undernutrition. CIAF captures nuances in undernutrition that may be missed by individual measures of stunting, underweight and wasting (Nandy & Miranda, 2008). Weights and heights were measured by NFHS‐4 trained staff members (two trained staff measured child length and height). For children less than 2 years of age, a SECA 417 Infantometer (SECA, Germany) was used to measure child length; for older children and adults, staff used a SECA 213 stadiometer to measure height. A SECA 874 U digital scale measured child and adult body weight (IIPS, 2014). Height was measured in metres and weight in kilogrammes. Implausible values were defined as 6 *SD* from the mean or more for HAZ and WAZ (Shi, Korsiak, & Roth, 2018).

### 2.4 Statistical analysis

Analyses consisted of (1) descriptive analyses of the distribution of covariates among children with stunting, underweight and CIAF; and (2) multivariable logistic regression to determine the odds ratios (ORs) for stunting, underweight and CIAF among children who consumed dairy milk compared with children who consumed no dairy milk.

Prevalence estimates of stunting, underweight and CIAF were calculated accounting for the survey design and sampling weights. Descriptive analyses using frequencies and proportions were conducted to quantify the distribution of covariates among individuals with stunting and underweight.

Unadjusted logistic regression was used to assess the relationship between dairy milk consumption (binary exposure) and ORs for stunting, underweight and CIAF (binary outcomes). Separate models were used for each outcome. Adjusted multivariable models included covariates determined a priori (listed above) to assess for potential confounding. Linear regression was used to determine the relationship between dairy milk consumption and HAZ and WAZ *z* scores. Coarsened exact matching (CEM) was used, which has been demonstrated to limit bias and confounding, reduce model dependence and improve estimates of directionality within relationships (Iacus & Porro, 2011). Using CEM, we created a cohort (*n* = 28,207) matched on age in months, diet score, state of residence, wealth quintile, maternal education, maternal BMI, birth weight, birth size and time of breastfeeding initiation after birth to gain an estimate of the directionality within the relationship between dairy milk consumption and child anthropometric outcomes.

Twenty states with the highest number of respondents were individually analysed for the relationship between dairy milk intake and odds of stunting, underweight and CIAF within each state. Additional stratified and interaction analyses were conducted according to high‐ or low‐milk consumption at the state level. We defined state level of consumption based on the median proportion across states (33%).

Models accounted for survey design characteristics and sampling weights using the survey package in R. Since NFHS‐4 was a two‐stage stratified cluster sample, weights were calculated for each stage and cluster to determine sampling probabilities for each (IIPS and ICF, 2017). For all statistical tests, an alpha level of 0.05 was used, and 95% confidence intervals were calculated. Multicollinearity was assessed using the variance inflation factor (VIF); all covariates remained under a VIF of 3.5 (O'Brien, 2007). All analyses were conducted using R version 3.5.1 (R Core Team, 2014).

The proposed study used publicly available and anonymised data obtained from the Demographic and Health Surveys programme (IIPS and ICF, 2017). Permission to access the data via online registration through the DHS website was obtained. This analysis involved secondary use of an anonymous public‐use health survey without access to identifiers. According to TCPS2, this research is considered exempt from REB review (Government of Canada, 2018).

### 2.5 Ethical considerations

This study was conducted according to the guidelines in the Declaration of Helsinki, and all procedures involving research study participants were approved by the International Institute for Population Sciences (Mumbai, India). Verbal informed consent was obtained from all participants of the National Family Health Survey. Verbal consent was witnessed and formally recorded. This research is based on a secondary analysis of a publicly available dataset without identifiers and was considered exempt from IRB approval under TCPS 2 (Tri‐Council Policy Statement: Ethical Conduct for Research Involving Humans).

## 3 RESULTS

**FIGURE 1:**
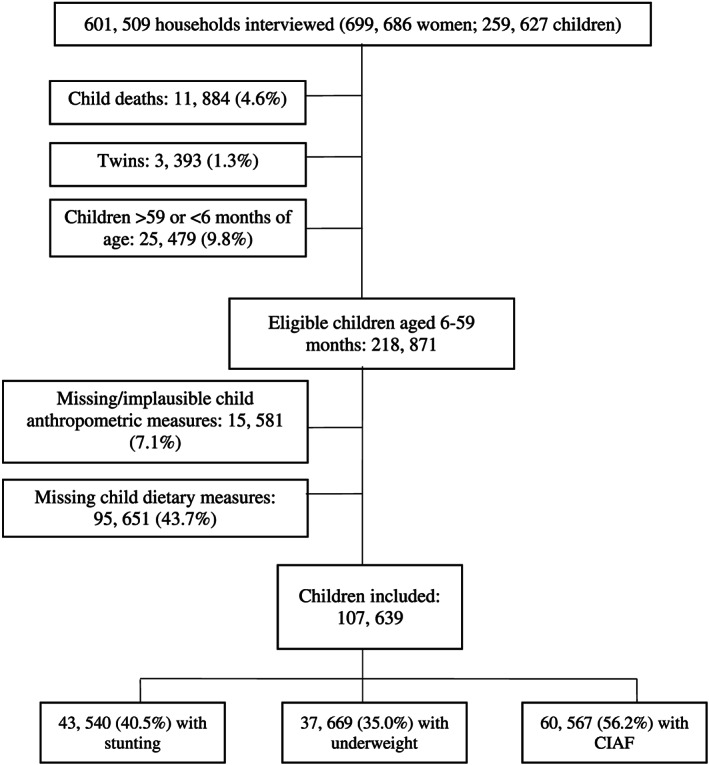
Participant flow diagram showing exclusions and final study population, 2015–2016 National Family Health Survey (NFHS), India

A total of 107,639 children aged 6–59 months were included in this analysis (Figure 1). Participant characteristics are shown in Tables 1 and 2. The mean age of children was 24.9 months, and 49% were male. Within the study sample, 40.5% of children had stunting, 35.0% had underweight and 56.2% had CIAF. At the time of the survey, 86.8% of children were currently breastfed. Children's dairy milk consumption appeared to be similarly distributed across wealth, dietary diversity and mother's education (Table S1).

**TABLE 1:** Characteristics of children aged 6–59 months living in India with caregivers who participated in the Nutrition and Family Health Survey (NFHS‐4) in 2015–2016

Age, months, mean (*SD*)	24.9 (15.3)
Sex, males (*n*, %)	52,703 (49.0)
HAZ, mean (*SD*)	−1.52 (1.73)
WAZ, mean (*SD*)	−1.52 (1.23)
Weight for height, mean (*SD*)	−0.95 (1.38)

Abbreviations: HAZ, height‐for‐age *z* score; *SD*, standard deviation; WAZ, weight‐for‐age *z* score.

**TABLE 2:** Child, household and maternal characteristics by anthropometric classification^a^

		Stunted		Underweight		Composite index of anthropometric failure (CIAF)	
Characteristic	*N* (%)	*n* (%)^b^	Prevalence^c^	*n* (%)^b^	Prevalence^c^	*n* (%)^b^	Prevalence^c^
All India	107,639	43,540 (40.5)	40.5	37,669 (35.0)	35.0	60,657 (56.2)	56.2
Child dietary diversity score (0–7 possible points)^d^							
Quintile 1 (poorest)	27,460 (25.5)	11,510 (26.4)	41.9	10,356 (27.5)	37.7	16,149 (26.6)	58.8
Quintile 2	21,272 (19.8)	8,635 (19.8)	40.6	7,994 (21.2)	37.6	12,314 (20.3)	57.9
Quintile 3	20,301 (18.9)	8,346 (19.2)	41.1	7,259 (19.3)	35.8	11,560 (19.1)	56.9
Quintile 4	26,829 (24.9)	6,664 (15.3)	24.8	5,523 (14.7)	20.6	9,045 (14.9)	33.7
Quintile 5 (highest)	11,777 (10.9)	8,385 (19.3)	71.1	6,537 (17.4)	55.5	11,499 (19.0)	97.6
Fever and/or cough in past 2 weeks (no)	88,365 (82.1)	36,263 (83.3)	41.0	31,163 (82.7)	35.3	50,132 (82.8)	56.7
Fully vaccinated (yes)	45,907 (41.9)	18,215 (41.8)	39.7	15,498 (41.1)	33.8	24,995 (41.3)	54.4
Vitamin A supplementation in past 6 months (yes)	58,438 (54.3)	23,116 (53.1)	40.0	20,091 (53.3)	34.4	32,448 (53.6)	55.5
Size of child at birth							
Larger than average	17,812 (16.6)	6,491 (14.9)	36.4	5,540 (14.7)	31.1	9,263 (15.3)	52.0
Average	75,122 (69.8)	30,030 (69.0)	40.0	25,651 (68.1)	34.1	41,881 (69.1)	55.8
Smaller than average	12,373 (11.5)	5,884 (13.5)	47.6	5,562 (14.8)	45.0	7,946 (13.1)	64.2
Birth weight (g), mean (*SD*)	2,829 (592)	2,751 (603)		2,696 (589)		2,755 (593)	
Currently breastfeeding (yes)	93,461 (86.8)	38,084 (87.5)	40.7	33,395 (88.9)	35.7	53,198 (87.8)	56.9
Breastfeeding initiation							
≥1 h of birth	58,077 (55.5)	24,047 (56.7)	41.4	21,148 (57.6)	36.4	33,318 (56.4)	57.3
<1 h of birth	46,625 (44.5)	18,356 (43.3)	39.4	15,597 (42.4)	33.5	25,719 (43.6)	55.2
Missing	2,937	1,137 (2.6)		924 (2.5)		1,530 (2.5)	
Maternal height (cm)							
<145	13,040 (12.1)	7,684 (17.7)	58.9	6,766 (18.0)	51.9	9,552 (15.8)	73.3
145–149.9	29,614 (27.5)	14,001 (32.2)	47.2	12,287 (32.7)	41.5	18,703 (30.9)	63.2
150–154.9	36,034 (33.5)	13,743 (31.6)	38.1	11,692 (31.1)	32.4	19,643 (32.5)	54.5
155–159.9	20,915 (19.5)	6,234 (14.3)	29.8	5,292 (14.1)	25.3	9,540 (15.8)	45.6
≥160	7,888 (7.3)	1,820 (4.2)	23.1	1,585 (4.2)	20.1	3,042 (5.0)	38.6
Maternal BMI (kg/m^2^) among non‐pregnant mothers							
<18.5	27,635 (27.7)	12,988 (32.7)	47.0	13,070 (38.0)	47.3	18,236 (32.8)	66.0
18.5–24.9	61,081 (61.3)	23,761 (59.9)	39.0	19,384 (56.3)	31.7	33,136 (59.6)	54.2
≥25.0	10,932 (11.0)	2,932 (7.4)	26.8	1,979 (5.7)	18.1	4,233 (7.6)	38.7
Mother's education							
No schooling	34,147 (31.7)	17,468 (40.1)	51.1	15,652 (41.6)	45.8	22,883 (37.8)	67.0
Primary	16,094 (15.0)	7,300 (16.8)	45.3	6,260 (16.6)	38.9	9,904 (16.4)	61.5
Secondary	48,131 (44.7)	16,681 (38.3)	34.7	14,124 (37.5)	29.3	24,307 (40.1)	50.5
Higher than secondary	9,267 (8.6)	2,091 (4.8)	22.6	1,633 (4.3)	17.6	3,473 (5.7)	37.5
Household wealth quintile^e^							
Quintile 1 (poorest)	30,045 (27.9)	15,806 (36.3)	52.6	14,597 (38.8)	48.6	20,920 (34.5)	69.6
Quintile 2	26,162 (24.3)	11,563 (26.6)	44.2	9,806 (26.0)	37.5	15,664 (25.9)	59.9
Quintile 3	21,570 (20.0)	8,029 (18.4)	37.2	6,662 (17.7)	30.9	11,408 (18.8)	52.9
Quintile 4	17,106 (15.9)	5,165 (11.9)	30.2	4,260 (11.3)	24.9	7,763 (12.8)	45.4
Quintile 5 (richest)	12,756 (11.9)	2,977 (6.8)	23.3	2,344 (6.2)	18.4	4,812 (7.9)	37.7
Improved drinking water source	89,812 (83.4)	36,592 (84.0)	40.7	31,618 (83.9)	35.2	50,662 (83.6)	56.4
Safe disposal of stools	56,868 (52.8)	20,911 (48.0)	36.8	17,140 (45.5)	30.1	29,242 (48.3)	51.4
Improved sanitary facility	48,407 (45.0)	15,918 (36.6)	32.9	12,600 (33.4)	26.0	22,893 (37.8)	47.3
Household air quality related to cooking fuels used							
Solid fuels	73,874 (72.6)	33,240 (80.3)	45.0	29,112 (81.6)	39.4	45,137 (78.6)	61.1
Clean fuels	27,905 (27.4)	8,136 (19.7)	29.2	6,581 (18.4)	23.6	12,302 (21.4)	44.1

Abbreviations: BMI, body mass index; *SD*, standard deviation.

^a^ Counts are unweighted; frequencies are adjusted for survey weights.

^b^ Column percentages.

^c^ Row percentages.

^d^ Diet diversity quintiles were created within the following age groups: <12; 12–24; 24–36; 36–48; and >48 months to account for age‐specific diet variation.

^e^ The wealth index and corresponding quintiles were created at the household level in the entire NFHS‐4 sample (Bassani, Corsi, Gaffey, & Barros, 2014); these were retained in the current sample and analysis.

Unadjusted logistic regression models showed that children who consumed dairy milk in the previous day or night had lower odds of underweight, stunting and CIAF. When adjusted for all covariates specified a priori (listed above), these relationships were maintained (Table 3). Children who consumed dairy milk had 0.95 the odds of underweight (95% CI 0.92–0.98, *P* = .0005), 0.93 the odds of stunting (95% CI 0.90–0.96, *P* < .0001) and 0.96 the odds of CIAF (95% CI 0.93–0.99, *P* = .004), compared with children who did not consume dairy milk after adjustment for all pre‐specified covariates. Analyses using CEM revealed weaker evidence of a relationship between dairy milk consumption and all anthropometric outcomes among children (Table S3).

**TABLE 3:** Logistic regression results showing the relationship between dairy milk intake and child anthropometric outcomes

	Unadjusted		Adjusted^a^	
	OR (95% CI)	*P* value	OR (95% CI)	*P* value
**Underweight**				
*Milk* (*yes*)	0.87 (0.86–0.89)	<.0001	0.95 (0.92–0.98)	.0005
**Stunting**				
*Milk* (*yes*)	0.92 (0.90–0.93)	<.0001	0.93 (0.90–0.96)	<.0001
**CIAF**				
*Milk* (*yes*)	0.93 (0.92–0.94)	<.0001	0.96 (0.93–0.99)	.004

*Note*: Covariate estimates not shown.

Abbreviations: CI, confidence interval; CIAF, composite index of anthropometric failure; OR, odds ratio.

^a^ Adjusted for age in months, diet score, wealth quintile, maternal education, maternal body mass index (BMI)^b^, birth weight, birth size, time of breastfeeding initiation after birth, current breastfeeding, fever or cough in past 2 weeks, home air quality related to cooking fuels used, access to an improved sanitary facility and drinking water source, unsafe disposal of stools, child vaccination status, vitamin A supplementation in the past 6 months and region of residence.

^b^ Stunting model was adjusted for maternal height in place of BMI.

Children who consumed dairy milk and resided in states with below‐median proportions of dairy milk consumption had lower odds of stunting, underweight and CIAF than those residing in states with higher dairy milk consumption (Table S2). There was evidence that children who consumed dairy milk had higher HAZ and WAZ scores, while adjusting for all pre‐specified covariates (Table 4).

**TABLE 4:** The relationship between dairy milk consumption and height‐for‐age *z* score (HAZ) and weight‐for‐age *z* score (WAZ)

	Coefficient (95% CI)	*P* value
**Height‐for‐age *z* score**		
*Milk* (*yes*)	0.07 (0.05–0.09)	<.0001
**Weight‐for‐age *z* score**		
*Milk* (*yes*)	0.05 (0.03–0.06)	<.0001

*Note*: Adjusted for age in months, diet score, wealth quintile, maternal education, maternal BMI (for WAZ model), maternal height (for HAZ model), birth weight, birth size, time of breastfeeding initiation after birth, current breastfeeding, fever or cough in past 2 weeks, home air quality related to cooking fuels used, access to an improved sanitary facility and drinking water source, unsafe disposal of stools, child vaccination status, vitamin A supplementation in the past 6 months and region of residence. Covariate estimates not shown.

## 4 DISCUSSION

In this large, population‐based and nationally representative survey from India, we investigated the cross‐sectional relationship between milk consumption and child stunting, underweight and anthropometric failure. We have several key findings. First, consumption of dairy milk among children aged 6–59 months was associated with slightly lower odds of stunting, underweight and anthropometric failure than those who did not. Second, this finding was relatively consistent across geographic regions, although probabilities were somewhat stronger in states with lower proportions of children who consumed dairy milk. Third, despite some attenuation in the relationship following matching with a reduced sample, direction and relative magnitude were maintained, which suggests adequate control of the known confounders. Despite differences in influential variables such as household wealth, region of residence, other dietary intake and maternal BMI, children who consumed dairy had more favourable growth than those who did not.

Results of the present study are consistent with other evidence showing potential for dairy milk consumption to support child growth worldwide (de Beer, 2012; Wiley, 2012). However, few other studies have evaluated the relationship between dairy milk consumption and child growth among young children in India. One analysis identified that Indian children younger than 2 years who consumed dairy milk had lower odds of stunting (Aguayo et al., 2016). A randomised controlled trial determined that consumption of vitamin‐ and mineral‐fortified milk among children aged 1–3 years in India reduced the burden of diarrhoea and acute respiratory illness and increased child height and weight relative to unfortified milk, suggesting that fortified milk may be an effective and acceptable strategy to reduce child morbidity in addition to providing macronutrients for growth (Sazawal et al., 2007).

There are a number of biological mechanisms that could be underlying the relationship between dairy consumption and child growth. Dairy intake increases circulating insulin‐like growth factor‐1 and is the only dietary source of whey and casein proteins, which are known to promote linear growth and may lower the incidence of stunting (Hoppe, Molgaard, & Michaelsen, 2006). Dairy milk is a nutrient‐dense food, providing carbohydrates, protein, fat, vitamin B_12_ and calcium, and is a vehicle for vitamin A and D supplementation (Michaelsen, Nielsen, Roos, Friis, & Mølgaard, 2011). It also contains highly bioavailable zinc, magnesium, potassium and phosphorous, which are essential for child development and especially important for catch‐up growth among children with anthropometric failure (Golden, 2009). It is probable that the combination of macro‐ and micronutrients provided by dairy contribute synergistically to child growth. We noted that the majority of children were currently breastfed. Dairy milk is richer in protein and micronutrients than breast milk, especially if the mother is undernourished. Although breast milk offers many physiological and immunological benefits, prolonged breastfeeding has been associated with lower maternal educational status and wealth, delayed introduction of complementary foods and lower weight gain and undernourishment among children in developing countries (Fawzi, Herrera, Nestel, el Amin, & Mohamed, 1998). Though dairy milk can be a complement to breast milk, it is possible that prolonged breastfeeding displaced dairy milk some children's diets.

Higher dietary diversity (defined as the inclusion of milk, meat, eggs, lentils, starchy staples, vitamin A fruits, other fruits and other dairy in the diet; Ruel & Menon, 2002) during childhood has been associated with lower risk of stunting and underweight (Corsi et al., 2016). Though dairy milk is relatively inexpensive and has become more accessible in India in recent years, it is possible that children who have access to dairy also may have access to other energy‐dense, growth promoting foods. Our findings suggest this may have been the case among children living in states with lower milk consumption, who had lower odds of anthropometric failure if they did have access to milk. Dairy milk consumption among Indian children can vary by maternal education and household income, with children of uneducated mothers and living in poor households consuming the least dairy products (Agrawal et al., 2019). However, India has steadily become the world's largest producer of dairy milk due to a rise in consumer demand and agricultural capacities (Gupta, 2015; National Institute of Nutrition, 2011). Fortified dairy products with vitamins A and D are now regulated in India (Food Safety and Standards Authority of India, 2019), which holds promise for improving child nutritional status provided they are safely handled, inexpensive and accessible to children. Though lactose intolerance affects a high proportion of South Asian adults, children are usually able to tolerate lactose well until adolescence or early adulthood (Heyman & Committee on N, 2006). Widespread, equitable access to dairy milk for children may be an important part of a dietary strategy to promote child growth in India. Fortified infant formula is also recommended for children when breast milk is unavailable (National Institute of Nutrition, 2011) and may play a similar role to dairy milk in promoting child growth; however, formula was not included in this analysis due to its distinct nutritional properties from dairy milk (Institute of Medicine [US] Committee on the Evaluation of the Addition of Ingredients New to Infant Formula, 2004).

This study had a number of strengths. A large, nationally representative survey that captured a wide range of exposures allowed for a high number of children to be included and control over many potentially confounding variables. The NFHS‐4 was the first survey to capture data from all 640 districts in India, providing comprehensive data on diverse households (IIPS and ICF, 2017). By using survey weights, we were able to account for non‐response and underrepresented groups in our sample (Package ‘survey’ [computer program], 2019). Use of CEM allowed for an estimate of directionality within the observed relationship by balancing covariate distributions between children who did and did not report consuming dairy milk (Iacus & Porro, 2011).

Limitations of this study include cross‐sectional design, which rendered our ability to describe a causal relationship between observed variables. Though our analysis accounted for variables related to diet and child growth such as maternal BMI, region and wealth, residual confounding is possible. We were not able to identify whether the milk consumed by children in this analysis was fortified with vitamins A and D or other nutrients; therefore, the mechanism at work remains unclear. Prospective cohort studies or randomised controlled trials are needed to further evaluate the effect of commercially available dairy milk on child health in India. Milk consumption was measured as a dichotomous variable at a single point in time, and volume was not quantified. The large sample size increases the likelihood of Type I error (rejecting a null hypothesis that is true) or of finding a difference that is statistically but not practically significant.

Anthropometric failure remains a persistent problem for children in India, such that over one‐third of children in India are stunted or underweight (IIPS and ICF, 2017). Among children aged 6–59 months in India who participated in the nationally representative NFHS‐4 survey, those who consumed dairy milk had lower odds of stunting, underweight and anthropometric failure than those who did not, after adjusting for relevant covariates. Given these results, dietary strategies in India may prioritise widespread and inexpensive access to sterile dairy milk to optimise child growth.

This research received no specific grant from any funding agency, commercial or not‐for‐profit sectors.

## CONFLICTS OF INTEREST

The authors declare that they have no conflicts of interest.

## CONTRIBUTIONS

SV and DC conceptualised the research question, designed and performed the statistical analysis and wrote the manuscript. Both authors approved the final manuscript as submitted.

## Supporting information


**Table S1.** Dairy milk consumption by wealth, dietary diversity, and mother's education.
**Table S2.** The relationship between dairy milk consumption and odds of stunting, underweight and CIAF in states with low vs. high dairy milk consumption.*
**Table S3.** Odds of underweight, stunting and CIAF among children matched using coarsened exact matching (CEM), for age in months, diet score, region of residence, wealth quintile, maternal education, maternal body mass index (BMI)**, birth weight, birth size, and time of breastfeeding initiation after birth. (*n = 28,207 children matched)*
