# Supplementary material for: Milk consumption and childhood anthropometric failure in India: Analysis of a national survey
Source: Matern Child Nutr. 2020 Sep 30;17(2):e13090. doi: 10.1111/mcn.13090 (PMC7988841; doi:10.1111/mcn.13090)
Supplement: Supplementary file 1 — Table S1. Dairy milk consumption by wealth, dietary diversity, and mother's education. Table S2. The relationship between dairy milk consumption and odds of stunting, underweight and CIAF in states with low vs. high dairy milk consumption.* Table S3. Odds of underweight, stunting and CIAF among children matched using coarsened exact matching (CEM), for age in months, diet score, region of residence, wealth quintile, maternal education, maternal body mass index (BMI)**, birth weight, birth size, and time of breastfeeding initiation after birth. (n = 28,207 children matched) [file MCN-17-e13090-s001.docx]

**Supplemental Material for** ***Milk consumption and childhood anthropometric failure in India: Analysis of a national survey***

**Supplemental Table 1.** Dairy milk consumption by wealth, dietary diversity, and mother’s education.

| **Dairy milk consumption** | **Yes (n= 39146)** | **No (n= 68493)** |
| --- | --- | --- |
| **Household wealth quintile** | | |
| **Quintile 1 (poorest)** | 7944 (20) | 22101 (32) |
| **Quintile 2** | 8673 (22) | 17489 (26) |
| **Quintile 3** | 8396 (21) | 13174 (19) |
| **Quintile 4** | 7514 (19) | 9592 (14) |
| **Quintile 5 (richest)** | 6619 (17) | 6137 (9) |
| **Dietary diversity score quintile** | | |
| **Quintile 1 (poorest)** | 17142 (44) | 40762 (60) |
| **Quintile 2** | 7967 (20) | 10884 (16) |
| **Quintile 3** | 9141 (23) | 11937 (17) |
| **Quintile 4** | 2032 (5) | 2293 (3) |
| **Quintile 5 (highest)** | 2864 (7) | 2617 (4) |
| **Mother’s education** | | |
| **No schooling** | 10613 (27) | 23534 (34) |
| **Primary** | 5360 (14) | 10734 (16) |
| **Secondary** | 18379 (47) | 29752 (43) |
| **Higher than secondary** | 4794 (12) | 4473 (7) |
| **Child age** | | |
| **6-12 months** | 7250 | 15358 |
| **12-24 months** | 18883 | 25770 |
| **24-36 months** | 3147 | 7590 |
| **36-48 months** | 5481 | 11032 |
| **48-59 months** | 4385 | 8743 |

All columns are presented as n (% of column total).

**Supplemental Table 2.** The relationship between dairy milk consumption and odds of stunting, underweight and CIAF in states with low vs. high dairy milk consumption.*

|  | **OR (95% CI)** | **p value** | **OR (95% CI)** | **p value** |
| --- | --- | --- | --- | --- |
| **Low dairy milk consumption** | | | **High dairy milk consumption** | |
| **Underweight** | | | | |
| *Milk (yes)* | 0.91 (0.87 to 0.96) | 0.0006 | 0.97 (0.94 to 1.00) | 0.06 |
| **Stunting** | | | | |
| *Milk (yes)* | 0.91 (0.87 to 0.96) | 0.0006 | 0.96 (0.93 to 0.99) | 0.01 |
| **CIAF** | | | | |
| *Milk (yes)* | 0.94 (0.89 to 0.98) | 0.01 | 0.96 (0.93 to 0.99) | 0.02 |

*The median proportion of children who consumed dairy milk among 36 states was 0.33. Regions were dichotomized into low and high consumption around the median. Models were adjusted for age in months, diet score, wealth quintile, and maternal education.

**Supplemental Table 3.** Odds of underweight, stunting and CIAF among children matched using coarsened exact matching (CEM), for age in months, diet score, region of residence, wealth quintile, maternal education, maternal body mass index (BMI)**, birth weight, birth size, and time of breastfeeding initiation after birth. (*n= 28,207 children matched)*

|  | **OR (95% CI)** | **p value** |
| --- | --- | --- |
| **Underweight** | | |
| *Milk (yes)* | 0.96 (0.91 to 1.01) | 0.15 |
| **Stunting** | | |
| *Milk (yes)* | 0.95 (0.90 to 1.00) | 0.07 |
| **CIAF** | | |
| *Milk (yes)* | 0.95 (0.90 to 1.01) | 0.09 |

**Stunting model was adjusted for maternal height in place of BMI.
